# On expert curation and scalability: UniProtKB/Swiss-Prot as a case study

**DOI:** 10.1093/bioinformatics/btx439

**Published:** 2017-07-13

**Authors:** Sylvain Poux, Cecilia N Arighi, Michele Magrane, Alex Bateman, Chih-Hsuan Wei, Zhiyong Lu, Emmanuel Boutet, Hema Bye-A-Jee, Maria Livia Famiglietti, Bernd Roechert, The UniProt Consortium

**Affiliations:** 1 Swiss-Prot Group, SIB Swiss Institute of Bioinformatics, Centre Medical Universitaire, Geneva 4, Switzerland; 2 Protein Information Resource, University of Delaware, Newark, DE, USA; 3 European Molecular Biology Laboratory, European Bioinformatics Institute (EMBL-EBI), Wellcome Genome Campus, Hinxton, Cambridge, UK; 4 National Center for Biotechnology Information (NCBI), US National Library of Medicine, Bethesda, MD, USA; 5 Protein Information Resource, Georgetown University Medical Center, Washington, DC, USA

## Abstract

**Motivation:**

Biological knowledgebases, such as UniProtKB/Swiss-Prot, constitute an essential component of daily scientific research by offering distilled, summarized and computable knowledge extracted from the literature by expert curators. While knowledgebases play an increasingly important role in the scientific community, their ability to keep up with the growth of biomedical literature is under scrutiny. Using UniProtKB/Swiss-Prot as a case study, we address this concern via multiple literature triage approaches.

**Results:**

With the assistance of the PubTator text-mining tool, we tagged more than 10 000 articles to assess the ratio of papers relevant for curation. We first show that curators read and evaluate many more papers than they curate, and that measuring the number of curated publications is insufficient to provide a complete picture as demonstrated by the fact that 8000–10 000 papers are curated in UniProt each year while curators evaluate 50 000–70 000 papers per year. We show that 90% of the papers in PubMed are out of the scope of UniProt, that a maximum of 2–3% of the papers indexed in PubMed each year are relevant for UniProt curation, and that, despite appearances, expert curation in UniProt is scalable.

**Availability and implementation:**

UniProt is freely available at http://www.uniprot.org/.

**Supplementary information:**

Supplementary data are available at *Bioinformatics* online.

## 1 Introduction

Biological knowledgebases have become indispensable for biomedical research by providing data in easily accessible formats. The Universal Protein Resource (UniProt) is one such key resource that acts as a central hub of protein knowledge by offering a unified view of protein sequence and functional information (UniProt, 2017). Expert curation constitutes a core activity of the UniProt Knowledgebase (UniProtKB) which is composed of two sections, UniProtKB/Swiss-Prot, the reviewed section containing expertly curated records with information extracted from the literature and curator-evaluated computational analysis, and UniProtKB/TrEMBL, the unreviewed section with computationally analyzed records, enriched with automatic annotation.

Bioinformatic predictions of protein function rely upon correctly annotated database sequences, and the presence of inaccurately or poorly annotated records introduces noise and bias to biological analyses (Bengtsson-Palme *et al.*,, 2016). Literature-based expertly curated data is highly reliable, and therefore considered the gold-standard, providing, in the case of UniProtKB, high-quality annotations for experimentally characterized proteins across diverse protein families (Keseler *et al.*, 2014; Schnoes *et al.*, 2009). In this way, it serves as a source of annotations that can be used for the development and enhancement of bioinformatics algorithms and text mining methods. In addition, literature-based annotations of characterized proteins are the basis for the automatic annotation of uncharacterized ones, a key challenge in the big data era which is witnessing the generation of large amounts of sequences (Oliver *et al.*, 2016; Pedruzzi *et al.*, 2015).

Despite the aforementioned needs and usage of expert curation, the question about its long-term sustainability has frequently been raised. Expert curation is considered to be a time-demanding and expensive activity (Bourne *et al.*, 2015). An important question raised by Bourne *et al.*, concerns the ability of expert curation to keep up with the continuing growth of the biomedical literature, with over 1 million papers published every year. The number of articles fully curated per year in UniProtKB/Swiss-Prot, ranging from 8000 to 10 000 articles per year based on the last 7 years, seems low in comparison to the amount of literature available, giving the impression that literature curation cannot scale in the face of the increasing amount of published papers. This picture is misleading because only publications that provide relevant information are included in UniProtKB/Swiss-Prot, while many articles that have been read or examined during the curation process are not included. However, we have not tracked this information in a formal manner until now.

To give a clearer picture of the landscape of curatable articles and to address concerns about the ability of literature-based curation to scale with the increase of biomedical literature, we performed a study of the ratio of relevant versus non-relevant papers. For this purpose, we used the PubTator text mining system (Wei *et al.*, 2013) to classify articles evaluated during the curation process. We monitored the literature triage process during a 6-month period with a set of curators in order to (i) determine the total number of articles that we read and/or evaluate, (ii) quantify the fraction of the relevant literature covered by UniProtKB and (iii) address the question of the scalability of expert curation in UniProtKB/Swiss-Prot.

## 2 Materials and methods

### 2.1 PubTator

**Fig. 1:**
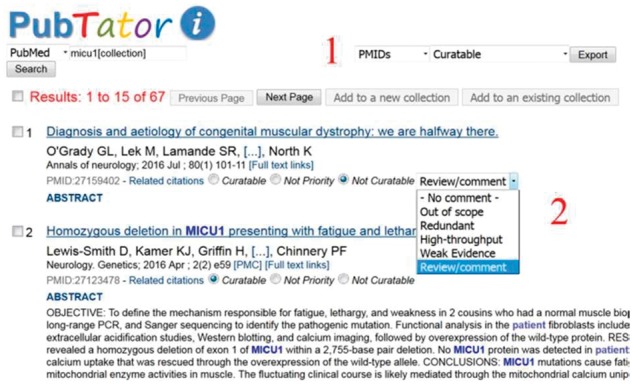
Screenshot of the PubTator tool. Some of PubTator’s functionalities include: (1) export of PubMed identifiers and annotations for the different sets (e.g. curatable and not-curatable); (2) menu for not-curatable options; access to abstract with annotations; and table with annotations and with links to UniProt accessions

PubTator (http://www.ncbi.nlm.nih.gov/bionlp/pubtator) is a web-based application that automatically annotates all articles in PubMed with key biological concepts via advanced text mining software tools (Wei *et al.*, 2013). To meet the specific needs of UniProt curation, a number of customizations were made to both the annotation results and user interface. First, all text-mined gene/protein annotations with corresponding NCBI Gene identifiers were converted to UniProt accessions. Next, we developed a frequency-based approach for ranking articles with rich protein information. We first added a third category for UniProt curators to classify an article—Not priority—in addition to ‘Curatable’ and ‘Not curatable’. Furthermore, five sub-categories were inserted under the existing ‘Not curatable’ category: ‘Out of scope’, ‘Redundant’, ‘High-throughput’, ‘Insufficient evidence’ and ‘Review/comment’ (Fig. 1).

### 2.2 Preparation of datasets

#### 2.2.1 Random sampling of 500 PubMed articles

To evaluate the proportion of PubMed articles that are relevant for UniProt curation, we first generated a set of 500 PubMed articles published from 2013 to 2015 (166 articles in 2013, and 167 in both 2014 and 2015) by random sampling.

#### 2.2.2 Weekly collection from selected journals

Each week, PubTator generates an update for new articles published in a selected set of relevant journals for protein research (Cell, Developmental Cell, Elife, Genes and Development, Molecular Cell, Nature Cell Biology, Nature Genetics, Nature, PLoS Biology, PLoS Genetics, Science, The EMBO Journal, The Plant Cell). All new articles are first mined for protein and species information and then ranked based on frequency of protein mentions.

#### 2.2.3 Protein-centric curation workflow

The UniProt curators selected for this analysis work in different annotation programs. E.B. is specialized in curation of plant proteins; H.B.-A.-J. is specialized in curation of *Caenorhabditis elegans* proteins; M.L.F. is specialized in curation of proteins associated with genetic diseases in human; B.R. is specialized in curation of vertebrate proteins; S.P. curates proteins across a variety of organisms.

The five UniProt curators first search PubTator for articles relevant to a specific protein (e.g. APC13 and Arabidopsis). PubTator shows the exact same search results as PubMed. Once an article title is clicked in the search results, PubTator directs the users to its curation page (aka abstract page) where the automatic computer pre-annotations can be examined (and revised). All the edits and comments are recorded in PubTator and can be downloaded, either in bulk or by single article, for further analysis.

## 3 Results

### 3.1 Classification of the published literature

The selection of relevant and accurate literature is a key factor in the expert curation process (Poux *et al.*, 2014). In UniProt, we do not aim to curate every available publication for a given protein. Instead, we concentrate curation efforts on publications that provide relevant novel information. For every paper included, there may be many other papers that are reviewed but excluded because they contain information that is redundant with existing literature-based annotations, provide weak evidence, are review articles or are simply beyond the scope of the information which UniProt captures. During the UniProtKB/Swiss-Prot literature triage process, evaluated articles can be classified into the following categories:

1. Curatable: For papers containing relevant information which are either selected for curation or are already present in UniProtKB/Swiss-Prot.
2. Not priority: For articles containing curatable information where the reported data are not considered high-priority so the papers were not selected for curation.
3. Not curatable: For articles that report information that is not relevant for inclusion in UniProtKB/Swiss-Prot. We further classify such articles into a number of subcategories:
  - Not curatable—Out of scope: for papers that are not in the field of proteins or which use a protein as a marker.
  - Not curatable—Redundant: for papers that describe relevant information which is already present in UniProtKB/Swiss-Prot.
  - Not curatable—High-throughput: for articles that report high-throughput studies, since these generate a higher rate of false positives than classical assays. A separate expert-driven analysis pipeline exists for the integration of the proteomics data (Breuza *et al.*, 2016).
  - Not curatable—Insufficient evidence: for publications that report results that are not supported by strong experimental evidence and would need additional experimental confirmation for inclusion.
  - Not curatable—Review/comment: for review and/or comment articles. Note that reviews that are not in the field of proteins were classified as ‘Not Curatable—Out of scope’.

Given these triage criteria, we performed a set of experiments using PubTator, a web-based text-mining tool in a PubMed-like environment, which provides access to all MEDLINE articles (Wei *et al.*, 2013). A number of text-mining approaches have been integrated into PubTator to identify key biological entities such as gene, protein and species names, which are conveniently highlighted in the interface, facilitating the literature triage process. The interface has been adapted to suit the UniProt triage in a number of ways including linking of protein mentions to UniProt accessions, capability to filter by organism and ability to create collections of references. Most importantly, the tool enables the assignment of articles to the categories described above (Fig. 1).

It is worth noting that the triage process is a two-step process, involving a quick scan of the articles to identify the potential set for curation, and a more in-depth evaluation of that set to identify the curatable papers**.** We used different approaches to evaluate the number of papers that are curatable.

### 3.2 Proportion of PubMed which is curatable

We first addressed the question of the fraction of PubMed that is relevant for inclusion into UniProtKB/Swiss-Prot by examining a random selection of papers from PubMed. We triaged a random sample of 500 PubMed articles published from 2013 to 2015 (Table 1). Our dataset is a representative set of PubMed as it has a similar overall distribution of articles based on indexed publication types in PubMed to the whole PubMed collection (Supplementary Table S1). To ensure the accuracy of our classification method, the 500 articles were evaluated independently by 2 different curators and articles were assigned to the same category in >99% of the cases.

**Table 1.** Random sampling of PubMed

Curatable	19
Not Priority	17
Not Curatable
Out of scope	452
Redundant	2
High-throughput	2
Insufficient evidence	5
Review/comment	3
Total	500

Surprisingly, only 38 articles contained information potentially suitable for curation in UniProt (the ‘Curatable’, ‘Not priority’ and ‘Not Curatable—Redundant’ categories). Of these, only 19 articles contained relevant information for curation in UniProtKB/Swiss-Prot. However, when compared with existing information in the corresponding entries, only 10 (2%) would remain for full curation. An additional nine papers provided interesting information but would not add essential knowledge or would constitute weak evidence for an annotation. For example, Carry *et al.* (2015) describe a selective inhibitor of aurora kinases, however aurora kinase entries are well annotated in UniProtKB and already describe selective inhibitors (UniProtKB O14965, Q96GD4 and O88445). 90% of the publications in this random set are completely outside the scope of UniProtKB.

### 3.3 Number of curatable papers in a subset of journals

In a second approach, we assessed the number of articles that are curatable for UniProtKB/Swiss-Prot in a subset of journals (Table 2). These journals (Cell, Developmental Cell, Elife, Genes and Development, Molecular Cell, Nature Cell Biology, Nature Genetics, Nature, PLoS Biology, PLoS Genetics, Science, The EMBO Journal, The Plant Cell) were selected based on their overall impact in the field combined with the fact that their content includes valuable information frequently prioritized for curation in UniProtKB/Swiss-Prot. PubTator automatically generates a weekly collection that includes the content of these journals. We have been using this tool to identify high-priority publications for curation for several years. During a six-month period, in addition to selecting articles for curation, we systematically classified all papers according to the criteria described in the previous section. We evaluated more than 5000 publications from the subset of journals described earlier. Again, the proportion of articles that are curatable is quite low: only 13.1% of articles indexed in PubMed for these journals constitute high-priority targets for curation in UniProtKB/Swiss-Prot. 65% of the publications are out of scope (Table 2). Around 10% of articles evaluated concern either reviews or high-throughput studies.

**Table 2.** Monitoring articles in a collection of journals

Curatable	659
Not Priority	681
Not Curatable
Out of scope	3259
Redundant	0
High-throughput	351
Insufficient evidence	32
Review/comment	331
Total	5013

### 3.4 Number of papers evaluated during the curation workflow

Finally, we tracked the number of publications that are evaluated during our routine protein-centric curation process, which typically starts with a literature search of a given protein. During a 6-month period, five curators systematically classified publications. To ensure that our analysis covered protein annotations from a variety of biological processes and a wide taxonomic range, we selected curators with different backgrounds and working in different annotation programs. During this period, these curators followed the classical UniProt expert curation workflow, a well-defined process that ensures that all records are handled in a consistent manner (Poux *et al.*, 2014), using PubTator to select and classify all publications evaluated during the curation process. In a 6-month period, more than 4500 papers were evaluated by these five curators (Table 3).

**Table 3.** Classification of papers evaluated during the expert curation process

Curatable	1398^a^
Not Priority	641
Not Curatable
Out of scope	1339
Redundant	385
High-throughput	159
Insufficient evidence	313
Review/comment	445
Total	4680

a Including 584 articles that were already present in UniProtKB/Swiss-Prot.

The proportion of papers that are curatable for UniProtKB/Swiss-Prot is low, even when specific terms such as gene or protein names are used to query the PubTator tool, as happens when curators perform searches during the curation process. Only 1398 out of 4680 articles evaluated (29.8%) were curatable, of which 584 were already present in UniProtKB/Swiss-Prot, meaning that only 814 new articles out of 4680 (17%) were relevant for curation. It is important to note that, throughout the 6-month period of the study, both the proportion of curatable papers and the proportion of articles already present in UniProtKB/Swiss-Prot were stable, reinforcing the accuracy of our analysis (Supplementary Table S2). Around a third of articles describe results that are out of the scope of UniProt, 10% of articles concern reviews and/or comment, and 8% of articles report redundant information, a frequent occurrence for papers reporting disease associations in human, where studies are made in different populations, generating a lot of redundant publications.

## 4 Discussion

### 4.1 A large fraction of papers indexed in PubMed are not relevant for UniProt curation

In UniProtKB/Swiss-Prot, knowledge is the driving factor for our expert curation effort and a targeted selection of papers is made in order to focus on publications that provide the maximum amount of high-quality information. Curators assimilate all the information from various sources, reconcile any conflicting results and compile the data into a concise but comprehensive report, which provides a complete overview of the information available about a particular protein (Poux *et al.*, 2014). Our literature triage clearly demonstrates that we evaluate a much higher number of articles than the 8000–10000 papers that we fully curate every year. Approximately only 17% of articles evaluated during the curation process are fully curated in UniProtKB/Swiss-Prot. When we extrapolate these results to the entire expert curation team, we estimate that we read or evaluate between 50 000 and 70 000 articles every year. In most cases, the abstract is sufficient to determine if an article is relevant for curation and the evaluation is fast. In other cases, it is necessary to read the full-text article, which takes more time. Measuring the number of curated publications is of course important, but it provides an incomplete picture of the complete set of papers evaluated during the curation process.

Our classification of articles during the triage process, however, should not be taken as a judgement on the quality of a publication. There can be different reasons why a publication is not selected for curation. Some excellent papers are not selected because the information relevant to UniProtKB is redundant with other publications present in the protein entry, while they contain outstanding information beyond the scope of UniProtKB. Moreover, our classification is not set in stone and can change: an article initially classified as ‘Not priority’ or ‘Not Curatable—Insufficient evidence’ may later be selected for curation when new data become available. A good example is provided by the DENND1B protein (UniProtKB Q6P3S1): an article reporting that variations in the DENND1B protein-coding gene are associated with susceptibility to asthma was not curated 4 years ago, because we considered that insufficient evidence was available at that time (Sleiman *et al.*, 2010). When the protein was updated in 2016, we revised our judgment based on new experimental results from other groups and reported this information from both articles (Sleiman *et al.*, 2010; Yang *et al.*, 2016).

Our analysis also shows that a large fraction of the published literature is not curatable for UniProtKB/Swiss-Prot purposes. Scientific literature is highly redundant. While redundancy is extremely useful for reproducibility of results, a key challenge in science, we prioritize new knowledge over information already described in an entry. For example, a total of 77 articles reported redundant information for spastin entry (UniProtKB Q9UBP0). We read the majority of these papers but decided not to add them in UniProtKB/Swiss-Prot since they do not provide any additional information. Many reviews and comments are also published: a total of 25 reviews were published for spastin. In many cases, we read such articles, but rarely integrate them because we favor curation of primary research results to allow for traceability of knowledge and so that curators can read the original research and make their own judgement on the data presented.

A large proportion of articles indexed in PubMed in the selection of journals that we parse every week are out of scope, even though these journals publish much valuable information for resources such as UniProtKB/Swiss-Prot. One reason for this is that articles that do not report experimental results, such as ‘news’ sections (Reardon, 2015) or corrections of previously published articles are all indexed in PubMed. Moreover, in general and multidisciplinary science journals, like Science and Nature, a lot of articles report on topics such as funding issues, political questions and climate change or publish articles not related to the life sciences (Hand, 2016). Last but not least, many articles describe new biological processes for which protein-coding genes have not yet been identified (Negishi *et al.*, 2016; Zimmerman *et al.*, 2016).

When applied to all journals indexed in PubMed, the proportion of articles that are out of the scope of UniProtKB is more significant: the random sampling shows that 90% of articles indexed in PubMed in recent years are not relevant or suitable for curation in UniProtKB/Swiss-Prot. As an example, >15% of the publications found in PubMed are not written in English, meaning that they will not be curated even if they are within the scope of UniProtKB data. Moreover, many publications report biomedical studies such as response to medication or prevalence of a disease in different populations.

Even for papers that are in the field of proteins, a substantial proportion is out of the scope of UniProtKB. For example, articles where MKI67/Ki-67 protein (UniProtKB P46013) is used as a marker of cell proliferation. More than 22,000 articles are indexed in PubMed concerning this protein, most of them using MKI67/Ki-67 as a marker, especially for comparing proliferation between tumor samples in the field of cancer research (Dowsett *et al.*, 2011; Richards-Taylor *et al.*, 2016). Ironically, while its function has been largely unclear for many years, recent results showed that its primary function is uncoupled from cell proliferation (Sobecki *et al.*, 2016). MKI67/Ki-67 is required to maintain dispersal of mitotic chromosomes by forming a steric and electrostatic charge barrier (Cuylen *et al.*, 2016).

For this study, we excluded proteins associated with thousands of articles, like MKI67/Ki-67, from the literature triage using PubTator, since proteins associated with thousands of articles of which only a small number are curatable for UniProtKB would have provided strongly skewed results.

### 4.2 Expert curation in UniProtKB is scalable

A major conclusion from the literature triage activity is that the proportion of publications relevant to UniProt curation is very small, hence expert curation can keep up with the increasing number of publications. The random sampling of PubMed shows that only 19 articles out of 500 contain curatable information, but that only 10 of them constitute high priority articles. Based on that, we estimate that a maximum of 2–3% of publications indexed in PubMed every year, between 20 000 and 25 000 articles, are curatable. In addition, the curation workflow experiment with a set of curators showed that 42% of the articles relevant for curation were already curated in UniProtKB/Swiss-Prot, while 58% concern not yet curated articles (Supplementary Table S2). The apparent big backlog should not be considered as such since, in that set of articles, the recently published articles may replicate data from older papers in the same set, rendering older papers redundant for the purposes of UniProtKB curation.

A number of steps were taken in our study in order to reduce bias. A wide range of proteins were curated, coming from a number of different organisms, such as human, mouse, *Arabidopsis thaliana*, *Caenorhabditis elegans* and *Escherichia coli*. Moreover, proteins involved in a wide variety of biological processes were curated, including DNA repair pathways, circadian cycles, cytoskeleton regulation, chromatin regulation, embryonic development, and flowering. Finally, our analysis concerned proteins newly integrated into UniProtKB/Swiss-Prot as well as updates of records already present in UniProtKB/Swiss-Prot.

To ensure that we capture the maximum amount of curatable information and do not overlook important publications, we have put in place robust mechanisms to identify proteins to curate. Part of the prioritization is performed using PubTator, by parsing the tables of content of a number of journals, but other mechanisms are also used. For example, we track newly identified 3D-structures. We also actively collaborate with other resources. For example, one of our curators is part of the Nomenclature Committee of the International Union of Biochemistry and Molecular Biology and we actively participate in the creation of new EC numbers and their curation. Our users also frequently point out when information is incomplete. All these mechanisms ensure that we identify and curate relevant proteins and do not accumulate a backlog of curatable papers.

We estimate that we curate 35–45% of PubMed that is relevant for UniProtKB/Swiss-Prot, so what about the part that we do not capture? For vertebrate proteins, in particular human, curation is quite comprehensive and up-to-date: all protein-coding genes from human are present in UniProtKB/Swiss-Prot and the proportion of articles relevant for curation already in UniProtKB/Swiss-Prot is higher compared with other organisms (>50% for human). We of course overlook articles, but most of the important ones are captured or will be captured in the coming years. Curation efforts are focused on recently published articles with the largest numbers of papers added for the current year or the previous year. For a number of organisms that are covered by the Model Organisms Databases (MODs), such as *Arabidopsis thaliana*, *Drosophila melanogaster* or *Caenorhabditis elegans*, we are not yet complete. Nevertheless, thanks to close collaborations with these resources we ensure that curation efforts are not duplicated, and UniProt data is supplemented with additional information curated by the MODs. It is also clear that the curation effort also reflects the size of research communities. We currently do not have sufficient resources to actively curate organisms studied by smaller scientific communities.

Our analysis demonstrates that expert curation in UniProt is able to keep up with biomedical literature for the organisms that are the main focus of curation and that the literature curation backlog is not as high as it first appears. This analysis is of course only relevant for UniProt, and only concerns the proportion of articles that are related to protein-coding genes in specific taxon groups. From this perspective, it would be interesting to perform similar studies in other resources of the ‘big data ecosystem’ described by Bourne *et al.* (2015). A recent article published by PomBase, showing that the number of articles published on *Schizosaccharomyces pombe* has been stable over the years and that curation of this body of data is sustainable, suggests that similar conclusions could be drawn for a number of MODs (Oliver *et al.*, 2016).

Our study also suggests that a reasonable increase in funding would allow us to cover the vast majority of relevant publications. The cost of expert curation is extremely modest, when compared with publication fees (Karp, 2016). Moreover, expert curation is highly accurate compared with other methods (Keseler *et al.*, 2014; Schnoes *et al.*, 2009). An independent survey assessing the value of biological database services concluded that the benefits to users and their funders are equivalent to more than 20 times the direct operational cost of the institute (http://www.ebi.ac.uk/about/news/press-releases/value-and-impact-of-the-european-bioinformatics-institute). This is why the common belief that expert curation is highly expensive and time-consuming is incorrect. From this perspective and from our analysis, expert curation should be considered as a major time-saver for the community for a very limited cost.

### 4.3 The need for expert biocurators

If the increase in the number of papers published every year has no major impact on the scalability of expert curation, it does affect the selection process, which is becoming a critical step in the curation workflow. A side effect of the increase in scientific publications concerns the growing presence of contradictory or incorrect results in the scientific literature. A number of articles have been published recently regarding the number of errors found in the scientific literature which are increasing to a level where science self-correction is no longer possible (Poux *et al.*, 2014; Sarewitz, 2016). One of these reported that >70% of researchers have tried and failed to reproduce another scientist's experiments, and more than half have failed to reproduce their own experiments (Baker, 2016; Santori, 2016). The presence of erroneous or irreproducible results in the scientific literature highly complicates the task of users and can affect interpretation of data.

Besides erroneous data, we have to take into consideration that knowledge is dynamic and that our understanding of biology continues to evolve as new experiments confirm or contradict previous results. When new findings invalidate previous ones, old curation is revisited in the light of new knowledge and annotation from previous papers re-evaluated. This is where expert curators are indispensable in providing an overview of the latest data in the context of previous findings.

Experienced curators with a strong background in wet lab research are adept at dealing with conflicting or erroneous information. In UniProtKB/Swiss-Prot, a curator will read and curate a number of publications from different groups in different organisms, helping to resolve conflicting issues and providing a general overview of the state of research in the field. This helps to ensure maximal efficiency when curating groups of related proteins by providing the in-depth background knowledge required, thus reducing the time taken for curation of each individual protein. UniProtKB/Swiss-Prot entries also provide biological background and context: when information is in contradiction with previous reports, it is clearly mentioned in the entry. For example, curation of the mitochondrial calcium uniporter in *Caenorhabditis elegans* (UniProtKB Q21121) following publication of its 3D-structure (Oxenoid *et al.*, 2016) generated a lot of collateral annotation: recent articles on the mitochondrial calcium uniporter were curated in different organisms, including human, mouse and *Dictyostelium discoideum* (UniProtKB Q8NE86, Q3UMR5, Q54LT0, respectively). Regulatory subunits of the mitochondrial calcium uniporter were also updated in order to provide a complete and up-to-date picture of the whole uniporter complex. This allowed the resolution of conflicting information, such as the topology of the SMDT1 regulatory subunit in human (UniProtKB Q9H4I9). Full curation of articles, regardless of the species, is also essential in light of new technologies: researchers use different organisms for their research and jump from *Caenorhabditis elegans* to human or non-model organisms in the same paper. The article describing the 3D-structure of mcu-1 in *Caenorhabditis elegans* (Oxenoid *et al.*, 2016) also contained experiments performed with the mitochondrial calcium uniporter in human (UniProtKB Q8NE86).

The growth of scientific literature strongly suggests that expert curation is needed more than ever to separate the wheat from the chaff and select articles that provide the maximum amount of reliable information that users need.

### 4.4 Improving efficiency of expert curation

Our results show that careful selection of papers is a critical step. The use of text-mining tools such as PubTator is of great help for curators by facilitating the literature triage process. Close collaboration between text-mining community and curators is essential for the continued improvement of text-mining accuracy and to save expert curation time. Moving forward, the data from the triage exercise performed in this work will be used to inform PubTator and tune the system to expedite the triage step in UniProt. Collaboration represents a major challenge for integration of text-mining tools in the biocuration workflow and their customization and maintenance. Many of the tools are developed as part of a research proposal and/or for proof of concept, in isolation from their potential users and not with the end goal of adoption. There is an ongoing effort in BioCreative to promote interaction between the biocuration and text-mining communities to lower these barriers (Wang *et al.*, 2016).

In addition, other initiatives could reduce the burden of expert curation. Structuring knowledge in scientific publications would provide benefits, and initiatives such as SourceData (http://sourcedata.embo.org) or Force11 (Bandrowski *et al.*, 2015) are very promising. Marking up the content of articles with controlled vocabularies that can be read by a machine would facilitate extraction of data from literature sources and save curation time. This would also help in improving accuracy of normalization for text-mining and also help to map papers and feed the additional bibliography section of entries for users looking for publications that have not been curated yet or have not been selected for curation. The presence of a structured format will however not resolve all problems and will be a long process because it is likely that only a subset of journals will adopt a structured format initially with the remainder containing free text information for an extended period. More importantly, the major challenge for users will still be the identification and selection of appropriate data and the extraction of reliable information.

Mechanisms will have to be found to filter knowledge and curators will continue to play a key role in this process. For these different reasons, we strongly believe that expertly curated databases are the cornerstone of scientific research. They provide gold-standard information for advancement of new methods, tools and algorithms, and allow users to keep up with the generation and evolution of knowledge, thus enabling new discoveries.

## Supplementary Material

Supplementary Data

Supplementary Data
