# Supplementary material for: On expert curation and scalability: UniProtKB/Swiss-Prot as a case study
Source: Bioinformatics. 2017 Jul 13;33(21):3454–60. doi: 10.1093/bioinformatics/btx439 (PMC5860168; doi:10.1093/bioinformatics/btx439)
Supplement: Supplementary Data [file btx439_supplement-table1-2.docx]

**Supplementary table 1.** Distribution of articles by publication types as indexed in Medline.

|  | **PubMed** | | **Random set** | |
| --- | --- | --- | --- | --- |
| **Total PMIDs** | 26398024 | Percent (%)^1^ | 494 | Percent (%) |
| **Journal articles** | 24761510 | 93.80 | 463 | 93.72 |
| **News** | 179312 | 0.68 | 3 | 0.61 |
| **Comment** | 671126 | 2.54 | 13 | 2.63 |
| **Biography** | 199909 | 0.76 | 2 | 0.40 |
| **Review** | 2152627 | 8.15 | 46 | 9.31 |
| **Published erratum** | 16833 | 0.06 | 3 | 0.61 |
| **English abstract^2^** | 1827927 | 7.38 | 23 | 4.66 |
| 1 Note that an abstract can belong to more than one category | | | | |
| 2 Full article not available in English | | | | |

**Supplementary table 2.** Proportion of curatable articles for the curation workflow analysis over time.

| Time | Total number of article evaluated | Curatable | | |
| --- | --- | --- | --- | --- |
|  |  | New article | Already present in UniProtKB/Swiss-Prot | Percent (%) of curatable papers already present in UniProtKB/Swiss-Prot |
| Month 1-2 | 2361 | 379 | 268 | 41,4 |
| Month 1-4 | 3986 | 646 | 480 | 42,6 |
| Month 1-6 | 4680 | 814 | 584 | 41,7 |
